# Effect of the Implementation of a Multiple-Behavior Self-Monitoring Intervention on Dietary Intake in Type 2 Diabetes: Secondary Data Analysis

**DOI:** 10.2196/49589

**Published:** 2024-08-20

**Authors:** Jisook Ko, Jing Wang, Ngozi Mbue, Susan Schembre, Stanley Cron

**Affiliations:** 1 UT Health San Antonio San Antonio, TX United States; 2 Florida State University Tallahassee, FL United States; 3 Texas Women's University Fort Sam Houston, TX United States; 4 Georgetown University Washington, DC United States; 5 The University of Texas Health Science Center at Houston Houston, TX United States

**Keywords:** electronic diary, technology-assisted self-monitoring, multiple-behavior intervention, type 2 diabetes, diabetes, self-monitoring, monitoring, dietary intake, monitor, carbohydrate intake, calories, education, diabetic, e-diary, e diary, self-care

## Abstract

**Background:**

An electronic diary embedded in a mobile device to monitor lifestyle can be as effective as traditional methods. However, the efficacy of self-monitoring multiple behaviors for dietary intake has not been well studied in people with diabetes.

**Objective:**

This study aimed to compare the effect of using technology-assisted self-monitoring versus paper diaries on changes in dietary intake.

**Methods:**

This is a secondary analysis of data collected from 39 people with type 2 diabetes as part of a 3-month pilot clinical trial. Changes in energy intake and the contribution of total fat intake and total carbohydrate intake to total calories (%) from baseline to after intervention (3 months) were evaluated.

**Results:**

In total, 26 (67%) of the 39 participants preferred mobile diaries over paper diaries. Participants in the mobile diary group showed slightly higher self-monitoring adherence. Linear mixed modeling results indicated a significant overall decrease in total energy intake (*P*=.005), dietary fat intake (*P*=.01), and carbohydrate intake (*P*=.08) from baseline to 3 months. No significant group differences were detected (*P*>.05).

**Conclusions:**

The implementation of a 3-month, multiple-behavior, self-monitoring intervention in Diabetes Self-Management Education programs has resulted in successful reduction in dietary intake (energy, fat, and carbohydrate), whichever self-monitoring method is chosen by participants according to their preferences. Long-term studies are needed to confirm our findings on dietary intake and examine other behavioral and disease outcomes that require monitoring.

## Introduction

Self-monitoring is an essential strategy used in behavioral lifestyle interventions for type 2 diabetes mellitus (T2DM) and obesity [1,2]. Diet, physical activity, weight, and blood glucose self-monitoring are effective in managing diabetes and preventing diabetes-related complications [3,4]. Targeting multiple behaviors rather than only single behaviors, self-monitoring allows the skills and knowledge learned for one behavior to be transferred to other behaviors [5], resulting in improvements across multiple behaviors [6]. In particular, individuals who would like to lose weight might be interested in sustaining diet and physical activity self-monitoring. Self-monitoring significantly mediates adherence to behavioral lifestyle interventions, including dietary behavior [7]. Traditionally, self-monitoring was performed using paper-based diaries. This paper-based method posed several challenges, including being time- and labor-intensive and requiring extensive numeracy and literacy skills, thus decreasing adherence to food logging over time and leading to low compliance [8]. Although more expensive than paper diaries, smartphones and mobile health devices became widely available, and self-monitoring using mobile devices became an appealing strategy supporting successful T2DM self-management [9,10]. This approach offers several advantages in enhancing the overall management of T2DM including enabling real-time monitoring, data accessibility to their self-monitoring data, behavioral change support, and patient engagement. In addition, self-monitoring using mobile devices reduces the burden and time effort of patients for recording daily activities related to T2DM self-management [9-11].

Most clinical trials designed to test the effect of a lifestyle intervention for T2DM randomly assigned the use of either a mobile diary or a paper-based method of self-monitoring; however, they rarely address patient preferences for how paper-based versus mobile diary methods influence outcomes of lifestyle intervention in T2DM. A recent study by our group found that an electronic diary embedded in a mobile device to monitor blood glucose can be as effective as the traditional method and is more likely to be used by participants than paper diaries [7]. However, no study has examined the efficacy of self-monitoring multiple behaviors on dietary behaviors.

Thus, we conducted a pilot study to test the feasibility of implementing a multiple-behavior self-monitoring intervention as an adjunct to a diabetes education program and tested the use of electronic or paper diaries to facilitate the patient self-monitoring process. The behavioral intervention used in this study was guided by the self-regulation theory, emphasizing the role of self-monitoring preceding self-awareness and self-regulation, and the social learning theory. This study aimed to examine the impact of the 3-month, multiple-behavior, self-monitoring intervention on patient dietary behaviors and whether using electronic diaries to facilitate self-monitoring would be more effective than paper diaries.

## Methods

### Design

This is a secondary analysis of data collected as part of a 3-month intervention implementation study. Study participants were offered the opportunity to self-monitor multiple health behaviors using either a mobile app for their smartphone or tablet (mobile diary) or a paper diary. This study aimed to (1) assess the preference for using a mobile diary over a paper diary and (2) compare the efficacy of using mobile versus paper diaries on changes in dietary intake. The primary outcomes of this study were changes in energy intake and the contribution of total fat intake and total carbohydrate intake to total calories (%) from baseline to after intervention (3 months). Secondary outcomes included changes in the consumption of sugars, dietary fiber, protein, and the total number of fruits and vegetables.

### Sample, Setting, and Recruitment

We posted flyers with information about the parent study at accredited Diabetes Self-Management Education programs at 3 partnering hospital systems or diabetes clinics in Houston, Texas. We invited those attending the Diabetes Self-Management Education group classes or individual visits to join the study before or after their sessions. Participants had the opportunity to speak with research staff about the study at the class site or could leave their contact information with the diabetes educators so research staff could contact them later.

Inclusion criteria for this study entailed (1) the ability to speak, read, and write English; (2) aged between 21 and 74 years; (3) having T2DM; (4) owning a smartphone compatible with the Jawbone UP24 fitness tracker (Jawbone Company); and (5) currently using a glucometer to monitor blood glucose levels. The exclusion criterion was current treatment for any severe psychiatric illness. We enrolled a total of 45 participants.

### Study Procedures

All participants who expressed an interest in participation completed an initial screening form. The screening form contained questions about study eligibility criteria and mobile phone use. Specifically, we asked participants whether they had a mobile phone, the make or model of the phone, and the type of phone plan (ie, data and SMS text messaging) that would support them in using the phone with the Jawbone app appropriately. We asked participants who met eligibility requirements to provide informed consent and provided questionnaire survey books to complete at home. Research personnel then scheduled a second meeting with the participant to collect the survey book, complete weekday and weekend dietary recalls over the phone or during the meeting, and deliver a single face-to-face intervention session.

### Intervention

Upon enrollment into the study, trained study staff provided participants with instructions on how to self-monitor multiple health behaviors for the duration of the parent intervention study. We modified the self-monitoring protocols from the Group Lifestyle Balance program and the Look AHEAD trial. Specifically, we provided participants with a digital scale to monitor their body weight daily, a pedometer to track the number of steps taken per day, measuring cups and a food scale to weigh their food and estimate portion size, and a behavioral lifestyle intervention guide with tips on how to improve their diets and increase their physical activity. Additionally, our trained interventionists educated participants on the contributions of diet and physical activity to energy balance as it relates to weight loss. With the interventionist, participants set weight loss goals ranging from 0.5 pounds per week to 2 pounds per week and specific dietary and physical activity goals. From these goals, a calorie allowance was determined based on the body weight, and the interventionist counseled the study participants on the impact of calorie, dietary fat, and carbohydrate intake on weight changes and blood glucose levels. Following the face-to-face session, research personnel followed up with each participant at 1 and 6 weeks to answer any participant questions regarding the instructions. The research team made themselves available to discuss questions and concerns on an ongoing basis throughout the 3-month study.

Depending on participant preference and smartphone ownership, we instructed participants to monitor their diet, physical activity, weight, and blood glucose daily. Participants had the option to self-monitor with a smartphone or a paper diary. We instructed smartphone owners who preferred to use their smartphone for self-monitoring to download 2 free smartphone apps: Lose It! (FitNow, Inc), with functions for logging diet, physical activity, and weight, and the Glucose Buddy (SkyHealth LLC), with functions for logging blood glucose. We selected apps that were free of charge and available on iOS and Android platforms. During the intervention session, participants received training on self-monitoring their dietary and physical activity habits and their weight and blood glucose using these 2 apps. Both apps track the foods by searching for foods in a food database, scanning the barcode on food labels, or snapping a picture (only Lose it!). Participants could constantly monitor their calorie intake through these apps.

We provided participants who owned a smartphone but preferred to self-monitor with a paper diary with the supplies to log their dietary and physical activity behaviors, weight, and blood glucose levels by hand. The study team designed the paper diaries to record the daily and weekly diet summary (eating time, portion size, calorie, fat gram, and carbohydrate content), physical activity (time, type, and duration of physical activity), weight, and blood glucose values. We provided participants using the paper diaries with an updated Calorie King book to estimate calorie and macronutrient content.

### Study Measures

#### Participant Characteristics

At baseline, we administered a sociodemographic questionnaire that captured participants’ age, gender, race, marital status, education, employment status, and years of having diabetes.

#### Dietary Intake

We assessed dietary intake at baseline and 3 months after intervention with the Automated Self-Administered 24-hour Dietary Recall-2014 (ASA24), which is freely available for use by researchers through the National Cancer Institute [12]. The ASA24 is based on a modified version of the interviewer-administered Automated Multiple Pass Method 24-hour Recall developed by the US Department of Agriculture [12-14]. The ASA24 was found reliable as the standard interviewer-administered dietary recall in collecting consumed foods with lower attrition rates [14,15]. Analytic output can be requested from the Researcher Website. Data dictionaries and samples of the output files are available for download from the Version 1 Researcher Website and the ASA24 Portal. We used this approach in the previous 2 studies. Trained research personnel (phone or face-to-face) interviewed participants at each study time point (baseline and after intervention) and 1 weekday and 1 weekend 24-hour recall. Research personnel entered the data directly into the ASA24 portal during the interview. We averaged the 2 days of recall data to represent the usual dietary intake at baseline and postintervention. We calculated the total energy intake (kcal) and the contribution of dietary fat, protein, and carbohydrates to total energy intake (%). We estimated the energy-adjusted intakes of sugar (g/1000 kcal) and dietary fiber (g/1000 kcal) and the total number of servings of fruit and vegetables.

### Sample Size

Using the effect size on weight loss from a self-monitoring focused intervention in obesity [13] and assuming 80% retention, we estimated that at least 40 participants (n=20 per group) were needed to detect a between-group (mobile vs paper diary) effect size of *d*=1.0 with 80% power.

### Statistical Analysis

We performed descriptive statistics on nutrition intakes for the mobile diary group and paper diary group on data collected at baseline and after intervention (3 months). We used repeated measures analysis with linear mixed models to compare the changes in nutrient intakes over time between the groups. Total energy intake, dietary fat, sugars, and the total number of fruits were nonnormally distributed and, thus, transformed to a natural log scale to better approximate a normal distribution. Statistical analyses were performed using SAS for Windows (version 9.4; SAS Institute).

### Ethical Considerations

The institutional review board approved the study at The University of Texas Health Science Center in Houston and partnering hospitals (application: HSC-SN-14-1027). Participants had to read and sign a consent form confirming that they were 18 years of age or older and that they consented to participate. All personal information was to be anonymized for data processing; hence, a participant identification number was required.

## Results

### Sample Demographics

We enrolled 45 participants from 3 partnering hospital systems or clinics in the 3-month study. At 3 months, only 39 participants completed the ASA24. Based on smartphone ownership and preferences, about 67% (n=26) were in the mobile diary group and 13 (33%) were in the paper diary group. Four participants were lost to follow-up between the baseline visit and the intervention session, and 2 were asked to withdraw from the study (1 for a medical reason and 1 for an unknown reason).

All study participants were 21 years or older (range 28-77 years), had T2DM, and used a glucometer to monitor their blood glucose levels. Table 1 presents the sociodemographic information and diabetes history.

**Table 1:** Demographic characteristics and diabetes history of the study sample (n=39).

Characteristic			Total (n=39)		Mobile diary (n=26)		Paper diary (n=13)	Chi-square or *t* test (*df*=38)	
Sex (female), n (%)			29 (74)		16 (62)		13 (100)	6.72^a,b^	
**Race (total: n=34, mobile diary: n=23; paper diary: n=11), n (%)**									4.49^b^
	Asian or Pacific Islander	4 (10)		1 (4)		3 (23)			
	Black or African American	13 (33)		8 (31)		5 (39)			
	White	15 (39)		13 (50)		2 (15)			
	Mixed race	7 (18)		4 (15)		3 (23)			
**Age (years)**									
	Mean (SD)	58.2 (11.4)		57.6 (12.2)		59.4 (9.8)		0.45^c^	
	Adults ≥60 years, n (%)	22 (57)		11 (42)		11 (85)		0.03^b^	
**Marital status, n (%)**									1.30^b^
	Currently married	17 (44)		13 (50)		4 (31)			
	Divorced or widowed or never married	22 (56)		13 (50)		9 (69)			
Years of formal education, mean (SD)			15.8 (3.4)		16.1 (3.4)		15.3 (3.3)	0.70^c^	
Employed full time, n (%)			23 (61)		15 (58)		8 (67)	0.28^b^	
Years living with diabetes, mean (SD)			11.3 (7.5)		11.9 (8.0)		9.8 (6.2)	0.68^c^	

^a^*P*<.05.

^b^Chi-square test.

^c^*t* test.

### Adherence to Self-Monitoring by the Number of Diaries Completed

Of the 39 participants (n=26, 67% for the mobile diary group and n=13, 33% for the paper diary group) who received the intervention, 68% (n=26) completed 2 dietary recalls (1 weekday and 1 weekend) at baseline and 3 months (Table 2). Although there were no significant differences in adherence to self-monitoring by the diary type (mobile diary vs paper diary), the total number of completed diaries was greater in the mobile diary group (mean 1.6, SD 1.0) compared to the paper diary group (mean 1.2, SD 0.6).

**Table 2:** Number of diaries completed over time (n=39).

Variable			Total (n=39)		Mobile diary (n=26)		Paper diary (n=13)		Chi-square or *t* test (*df*=38)	
**Frequency of completeness of diaries, n (%)**										5.92^a^
	2 weekdays and 2 weekends (baseline and 3 months)	26 (68)		15 (60)		11 (85)				
	1 weekday and 1 weekend (baseline only)	8 (21)		7 (28)		1 (8)				
	1 weekday and 1 weekend (3 months only)	1 (3)		0 (0)		1 (8)				
	2 weekdays and 1 weekend (mixed)	3 (8)		3 (12)		0 (0)				
Number of completed diaries, mean (SD)			1.5 (0.9)		1.6 (1.0)		1.2 (0.6)		1.36^b^	

^a^Chi-square test.

^b^*t* test.

### Nutrition Intake Outcomes

Dietary intake data are presented in Table 3. Initially, participants using the mobile diary reported consuming an average total energy intake of 1814.3 (SD 753.9; range 501.6-3930.9) kcal, whereas those using the paper diary reported an average of 1664.1 (SD 845.1; range 505.4-5536.9) kcal. The average total energy intake was significantly higher in the mobile diary group compared to the paper diary group (t_38_=7.39; *P*=.05). At the end of the 3-month intervention, participants in the mobile diary group reported an average of 1664.0 (SD 563.4; range 724.6-3336.4) kcal of total energy intake, while those in the paper diary group reported an average of 1093.4 (SD 753.9; range 259.7-2399.0) kcal. The data indicate that dietary fat, carbohydrates, and sugar lower after the intervention compared with baseline (Table 3).

**Table 3:** Descriptive and comparisons of dietary nutrients at baseline and 3 months by group.

Nutrients	Mobile diary		Paper diary		*P* value^a,b^	*P* value^c^
	Baseline	3 Months	Baseline	3 Months		
Energy (kcal), n (%)^d^	1814.3 (100)	1664.1 (100)	1664.1 (100)	1093.4 (100)	.*005*	.08
Dietary fat (% of total intake), n (%)	84.9 (24.7)	72.4 (22.6)	73.8 (22.4)	49.0 (22.4)	.*01*	.39
Carbohydrates (% of total intake), n (%)	188.6 (53.5)	178.8 (54.7)	180.8 (54.)	108.1 (51.2)	.*008*	.13
Protein (% of total intake), n (%)	73.0 (21.8)	73.6 (22.7)	71.6 (23.6)	58.5 (26.4)	.33	.33
Dietary fiber (g/1000 kcal), mean (SD)	7.7 (2.6)	8.8 (4)	8.9 (5.4)	10.5 (8.1)	.09	.18
Sugars (g/1000 kcal), mean (SD)	37.2 (23.9)	41.5 (26.6)	44.6 (28.8)	32.6 (22.0)	.*04*	.06
Total vegetables, mean (SD)^e^	1.3 (1.1)	1.1 (0.8)	1.4 (1.1)	1.2 (0.8)	.23	.91
Total fruits, mean (SD)^e^	0.7 (1.1)	0.9 (1.1)	1.1 (1.8)	0.5 (0.7)	.30	.28

^a^Before and after intervention comparison regardless of group.

^b^The italic values indicate that dietary fat, carbohydrates, and sugar lower after intervention compared with baseline.

^c^Between groups change over time.

^d^% is the contribution to total energy intake.

^e^Cup equivalent.

Linear mixed modeling results showed a significant overall decrease in total energy intake (*P*=.005), dietary fat intake (*P*=.01), carbohydrate intake (*P*=.008), and sugar intake (*P*=.04) from baseline to 3 months. However, we detected no significant group differences (*P*>.05). The results for the interaction of group and visits (*P*=.08) for total energy intake indicated a nonsignificant trend for a greater decrease between visits for the paper group. The results of the analyses on total sugar intake show a significant trend for an overall decrease in sugar intake (*P*=.04) and a nonsignificant trend for a more significant decrease in sugars reported between visits for the paper group (*P*=.06). The results of protein, fibers, total vegetables, and total fruit did not show significant differences between the 2 groups or between study visits.

## Discussion

### Principal Findings

Traditionally, behavioral lifestyle interventions incorporating self-monitoring as a behavior change technique use paper diaries to record multiple health behaviors. Advancements in smartphone technology with freely available mobile apps now enable self-monitoring. A few trials examining behavior changes using smartphone apps to self-monitor lifestyle modification enhanced behavioral weight loss [16,17], diabetic outcomes [10], and metabolic outcomes [18]. However, the efficacy of smartphone apps versus paper diaries has not been tested in the context of consideration for user preference in the type of dietary self-monitoring diaries. As such, this 3-month behavioral lifestyle intervention study implemented in a diabetes education program comparatively examined the effects of smartphone apps and paper diaries to self-monitor multiple lifestyle behaviors on changes in selected dietary outcomes.

Our demographic analyses indicated that participants were primarily female (n=29/39, 74.4%) and older adults aged 60 years or older (n=22/39, 56.5%). Most of the study participants (n=26/39, 66.7%) preferred using a mobile diary, including smartphone apps, for self-monitoring multiple behaviors. Current literature reports that older adults use their smartphones primarily for retrieving information or using it as a classic phone [19]; however, this study demonstrated a relatively high preference for using a smartphone among older adults (n=11, 55%). We found general acceptance for the mobile diary apps but observed sex differences. Female participants were more likely to use a paper diary, while male participants preferred a mobile diary. There was a lack of awareness of mobile diary apps among female participants and a lack of interest by older female participants.

Compared to paper diaries, participants who used smartphone apps for self-monitoring showed slightly higher adherence to self-monitoring through the number of completed diaries. Given that little is known about the extent to which people adhere over time, the study used the number of diaries completed over a 3-month period to measure self-monitoring adherence. Assessment bias is one of the methodological challenges in using self-monitoring in behavioral lifestyle interventions due to the lack of defined self-monitoring adherence [17]. The use of technology and electronic devices that date- and timestamp the self-monitoring behavior (the diary entry) provides an objective validation of these self-reported behaviors [7,20]. Previous studies consistently demonstrate that mobile and web-based health apps increased adherence and promoted behavior changes [21,22]. However, we found no significant findings for protein, fiber, total fruit, and vegetable intake. This study is the first attempt in a sample of patients with T2DM to examine the use of multiple-behavior self-monitoring on nutritional outcomes. Previous researchers focused on only obese populations and included diet and physical activity self-monitoring as part of the intervention. These findings suggest that smartphone apps are as effective as paper diaries in facilitating self-monitoring of multiple health behaviors in diabetes management interventions.

There are several study limitations. First, the sample had well-controlled T2DM, which may not represent the general diabetes population with comorbid overweight or obesity. Second, due to the small sample size and the self-reported data collection, further studies with larger sample sizes are needed to improve the validity and generalizability of the results. Third, participants indeed self-selected their groups, so it may produce the differences due to allocation bias, and not necessarily due to mobile versus paper intervention. Therefore, it needs caution in generalizing the results, and further research with a randomized design is warranted to establish a more robust causal relationship between the intervention type (mobile vs paper) and the observed outcomes. Finally, we used the ASA24 during a phone interview recall rather than participants performing the recall with the web-based format, which deviates from the original intent of ASA24; however, we determined that using the ASA 24 during a phone interview was a more robust way to collect dietary recall to improve accuracy and validity of dietary recall assessment.

### Conclusions

Implementing a 3-month, multiple-behavior, self-monitoring intervention in Diabetes Self-Management Education programs resulted in meaningful dietary intake on energy, fat, and carbohydrate intake, using whichever self-monitoring method participants chose according to their preferences. Long-term studies are needed to confirm our findings on dietary intake and examine other behavioral and disease outcomes that require monitoring.

## Abbreviations

ASA24: Automated Self-Administered 24-hour Dietary Recall-2014
T2DM: type 2 diabetes mellitus
